# Factors associated with family physicians’ perceived self-efficacy in multimorbidity management

**DOI:** 10.3389/fmed.2025.1596652

**Published:** 2025-06-06

**Authors:** Filipe Prazeres, Andreia Teixeira

**Affiliations:** ^1^Faculty of Health Sciences, University of Beira Interior, Covilhã, Portugal; ^2^Family Health Unit Beira Ria, Gafanha da Nazaré, Portugal; ^3^RISE-Health, Department of Community Medicine, Information and Health Decision Sciences, Faculty of Medicine, University of Porto, Porto, Portugal; ^4^MEDCIDS, Department of Community Medicine, Information and Health Decision Sciences, Faculty of Medicine, University of Porto, Porto, Portugal; ^5^AdiT-LAB, Instituto Politécnico de Viana do Castelo, Viana do Castelo, Portugal

**Keywords:** multimorbidity, primary care, self-efficacy, work-family conflict (WFC), perceived organizational support (POS), physician burden

## Abstract

**Background:**

Multimorbidity is a significant challenge for primary care. No previous research has examined self-efficacy in managing patients with multimorbidity among Portuguese family physicians.

**Aims:**

This study aims to assess self-efficacy levels in family physicians and identifying significant associations.

**Methods:**

Analytical cross-sectional study conducted among Portuguese family physicians from June to August 2024. A non-probability snowball sampling method was used to distribute a web-based survey. Ten independent variables (sex, age, marital status, children, professional stage, years of experience, workplace, work-family conflict, perceived organizational support and physician burden) were studied with the outcome variable – perceived self-efficacy. Multiple logistic regression model was performed.

**Results:**

102 family physicians completed the online questionnaire, with a median age of 38 years and a median work experience of 10.5 years. The majority of the sample were female (78.4%), married/cohabiting (70.6%), and employed in family health units (87.3%). Sixty-nine participants (67.6%) perceived their self-efficacy in multimorbidity management as high (Likert scale ratings 4 or 5). In the multivariate analysis being single, divorced, or widowed; having children; being a family physician trainee; and experiencing physician burden were associated with a reduced odds of perceived self-efficacy in managing multimorbidity.

**Conclusion:**

The findings of the present study highlight the importance of addressing physician burden to improve perceived self-efficacy in managing multimorbidity. Therefore, efforts should focus on reducing this burden by alleviating workplace stress and providing targeted training in managing multimorbidity. Improving self-efficacy is expected to encourage physicians to engage in proactive, patient-centered care, leading to better health outcomes.

## 1 Introduction

The World Health Organization (WHO) recognizes multimorbidity – the coexistence of two or more long-term health conditions in an individual (1) – as a significant challenge for primary care (2). It is associated with adverse drug events, intervention complications, care failures, inappropriate prescribing, medication overuse or underuse, non-adherence to treatment, and delayed diagnosis (2). These challenges are expected to increase with aging populations and the rising prevalence of long-term health conditions (2).

The Academy of Medical Sciences considers multimorbidity as a global health challenge and a priority for health research (3). A recently published systematic review and meta-analysis found that the global prevalence of multimorbidity is 37.2%, with Europe reporting a slightly higher prevalence of 39.2% (4). In the Portuguese population, the prevalence of multimorbidity is 38.3% (5), reaching 72.7% in the primary care setting (6).

Managing the complex issues of multimorbidity requires continuous care from family physicians and primary care teams. Family physicians face numerous challenges, including disorganization and fragmentation of healthcare systems, inadequacies in guidelines and evidence-based medicine, difficulties in delivering patient-centered care, and barriers to shared decision-making, as highlighted by Sinnot et al. (7) and more recently validated by Damarell et al. (8). Additionally, the strain of managing patients with multimorbidity, along with feelings of inadequacy in providing effective care (9), puts family physicians at risk of poor personal outcomes, such as burnout or leaving the profession entirely (8). Studying family physicians’ burden in managing patients with multimorbidity is essential, as it affects healthcare quality and physician well-being, with some authors even questioning whether complex cases should be managed by family physicians (10).

Perceived self-efficacy refers to individuals’ beliefs in their ability to achieve desired outcomes (11). Bandura stated that those with strong confidence in their capabilities view challenging tasks as opportunities to master rather than threats to avoid (11). This perspective encourages personal achievement, reduces stress, decreases susceptibility to depression (11), and enhances the quality of care in the healthcare context (12).

Among physicians, self-efficacy was found to have a significant negative relationship with burnout, emotional exhaustion, and depersonalization (13, 14). Furthermore, it positively impacts patient-centered care (15), well-being, work performance (16), job satisfaction and professional commitment among healthcare professionals (17, 18). It is also associated with seeking resources, embracing challenges, optimizing job demands, and reducing task delegation (19).

Previous research has indicated that self-efficacy is improved by perceived organizational support (20) – defined as beliefs about how much the organization values employees’ contributions and cares about their well-being (21) – including in the context of managing patients with multimorbidity (22). However, self-efficacy is hindered by work-family conflict (22) – defined as a type of interrole conflict where the demands of work and family are mutually incompatible in some way (23) – high stress levels, and burnout (24).

No previous research has examined self-efficacy in managing patients with multimorbidity among Portuguese family physicians. This study seeks to address this gap by assessing self-efficacy levels in family physicians and identifying significant associations.

## 2 Materials and methods

An analytical cross-sectional study was conducted among Portuguese family physicians from June to August 2024. Eligible participants included all family physicians working in primary healthcare in Portugal during the study period who were willing to participate and able to communicate in Portuguese. No specific exclusion criteria were established.

A non-probability snowball sampling method was used to distribute a web-based survey; the link to the electronic questionnaire, hosted on Google Forms, accompanied by an explicative letter was shared via institutional mailing lists and relevant medical groups on social media. Additionally, invited participants were encouraged to share the online questionnaire with their own contacts.

A pretest was conducted on 12 family physicians to evaluate the usability and technical functionality of the electronic questionnaire. No changes were needed following the pretest. The average completion time per participant was 10 min. Data collection was supported by two reminders sent during the study period, and only fully completed questionnaires were included in the data analysis, as all questions were mandatory in the Google Forms questionnaire.

Ethics approval was obtained from the University of Beira Interior Ethics Committee. All procedures followed in this study adhered to the principles of the Declaration of Helsinki. Informed consent was obtained from all participants, and their responses to the questionnaire were submitted anonymously.

The web-based questionnaire included sections on sociodemographic information, perceived self-efficacy, work-family conflict, perceived organizational support, and physician burden.

Perceived self-efficacy in multimorbidity management was assessed by using a 5-point Likert scale question: “How confident are you in providing optimal healthcare for patients with multimorbidity (coexistence of 2 or more chronic conditions)?” (1 = no confidence, 5 = very confident), as previously used in the literature (22).

Work-family conflict was measured using the 3-item work-family conflict scale developed by Matthews et al. (25) to assess participants’ agreement with three statements about the relationship between their work and family. This scale is an abbreviated version of Carlson et al. (26) work-family conflict scale previously validated in Portuguese (27). Participants rated statements such as: “I have to miss family activities due to the amount of time I must spend on work responsibilities”, on a 5-point Likert scale (1 = strongly disagree, 5 = strongly agree). Higher scores on the scale indicate higher levels of work-family conflict. The Cronbach’s alpha for this scale in the current sample was 0.775, indicating acceptable internal consistency reliability (28).

Perceived organizational support was measured using a shortened 8-item version of the Survey of Perceived Organizational Support (21), previously validated in Portuguese (29), by selecting the items with the highest factor loadings in Eisenberger et al.’s (21) analysis. The scale used a 7-point Likert format, ranging from 1 = strongly disagree to 7 = strongly agree. An example item is: “My organization really cares about my well-being.” Higher scores indicate a greater perception of organizational support. Items 2, 3, 5, and 7 were reverse-scored. The Cronbach’s alpha for this scale in the present study was 0.932, indicating good internal consistency reliability (28).

Physician burden was assessed by the Portuguese Questionnaire of Evaluation of Burden of Management of Multimorbidity in General and Family Medicine (SoGeMM-MGF) (30). The SoGeMM-MGF consists of 16 items, each rated on a 5-point Likert scale from 1 to 5. The total score is the sum of all 16 items, with higher scores indicating a greater perceived burden. An example item is: “Does dealing with patients with multimorbidity reduce the time available for other patients?” The Cronbach’s alpha for this scale in the present study was 0.779, indicating acceptable internal consistency reliability (28).

The data were analyzed using SPSS v. 28^®^ and Jamovi. Categorical variables were presented by absolute and relative frequencies, *n* (%). Normally distributed continuous variables were described by mean and standard deviation, M (SD), and non-normally distributed variables by median and interquartile range, Med (Q1; Q3). The normality of distributions was assessed by graphical observation of their histograms. In order to determine which of the 10 independent variables (sex, age, marital status, children, professional stage, years of experience, workplace, work-family conflict, perceived organizational support and physician burden) were associated with the outcome variable (perceived self-efficacy), simple logistic regressions were performed. The results of the logistic regressions were presented by the odds ratio (OR), the respective 95% confidence interval (CI 95%), and the *p*-value.

To perform multiple regressions, the minimum sample size should never be less than 30. If the number of independent variables is k, the minimum sample size should be 5k. Thus, in this study, the minimum sample size should be 50.

All variables that obtained a *p*-value less than 0.20 were included in a multiple logistic regression model. Subsequently, variables were excluded one by one, in descending order of *p*-value, until a final model was obtained with only significant variables. The adequacy of the final model was tested by the Hosmer-Lemeshow test.

*P*-values less than 0.05 were considered significant.

## 3 Results

Table 1 presents the characteristics of the 102 family physicians who completed the online questionnaire, with a median age of 38 years and a median work experience of 10.5 years. The majority of the sample were female (78.4%), married/cohabiting (70.6%), and employed in family health units (87.3%).

**TABLE 1:** Characteristics of the family physicians (*n* = 102).

Characteristics	*n* = 102
**Sex, *n* (%)**	
Male	22 (21.6)
Female	80 (78.4)
**Age, med [Q_1_; Q_3_]**	38 [31.75; 46]
**Marital status, *n* (%)**	
Married/cohabiting	72 (70.6)
Single/divorced/widowed	30 (29.4)
**Children, *n* (%)**	
No	54 (52.9)
Yes	48 (47.1)
**Professional stage, *n* (%)**	
Family physician trainee	28 (27.5)
Family physician specialist	74 (72.5)
**Years of experience, med [Q_1_; Q_3_]**	10.5 [5; 18]
**Workplace, *n* (%)**	
Family health units	89 (87.3)
Other primary care units	13 (12.7)
**Perceived self-efficacy, *n* (%)**	
1	2 (2.0)
2	2 (2.0)
3	29 (28.4)
4	60 (58.8)
5	9 (8.8)
**Work-family conflict, M (SD), min-max**	10.1 (2.8), 3–15
**Perceived organizational support, M (SD), min-max**	28.5 (11.6), 8–51
**Physician burden, M (SD), min-max**	61.8 (6.5), 42–76

Sixty-nine participants (67.6%) perceived their self-efficacy in multimorbidity management as high (Likert scale ratings of 4 or 5). Participants’ work-family conflict had a mean score of 10.1 (SD = 2.8), perceived organizational support had a mean of 28.5 (SD = 11.6), and physician burden had a mean of 61.8 (SD = 6.5).

Table 2 shows that, in the univariate logistic analysis of perceived self-efficacy in multimorbidity management, both increasing age and years of experience as a physician were associated with a higher odds of perceived self-efficacy. Additionally, family physicians with children were marginally more likely to report higher perceived self-efficacy than those without children (at a level of 0.20). On the other hand, being single, divorced, widowed, or being a family physician trainee, as well as experiencing work-family conflict and physician burden, were associated with a reduced odds of perceived self-efficacy in multimorbidity management.

**TABLE 2:** Univariate logistic regression associations with perceived self-efficacy in multimorbidity management.

	OR [CI 95%]	*p*-value
**Sex**		
Male	Ref	
Female	0.97 [0.35; 2.67]	0.952
**Age**	1.05 [1.00; 1.09]	0.050
**Marital status**		
Married/cohabiting	Ref	
Single/divorced/widowed	0.27 [0.11; 0.67]	0.004
**Children**		
No	Ref	
Yes	1.91 [0.81; 4.48]	0.137
**Professional stage**		
Family physician specialist	Ref	
Family physician trainee	0.06 [0.02; 0.19]	<0.001
**Years of experience**	1.05 [1.00; 1.10]	0.035
**Workplace**		
Family health units	Ref	
Other primary care units	1.69 [0.43; 6.62]	0.448
**Work-family conflict**	0.86 [0.74; 1.01]	0.058
**Perceived organizational support**	1.00 [0.96; 1.03]	0.895
**Physician burden**	0.87 [0.80; 0.94]	<0.001

In the multivariate analysis (Table 3), age, years of experience as a physician, and work-family conflict were not significantly associated with perceived self-efficacy. However, being single, divorced, or widowed; having children; being a family physician trainee; and experiencing physician burden were associated with a reduced odds of perceived self-efficacy in managing multimorbidity.

**TABLE 3:** Multivariate logistic regression associations with perceived self-efficacy in multimorbidity management.

	Multivariate logistic regression—initial model		Multivariate logistic regression—final model	
	**OR [CI 95%]**	***p*-value**	**OR [CI 95%]**	***p*-value**
**Marital status**				
Married/cohabiting	Ref		Ref	
Single/divorced/widowed	0.16 [0.03; 0.86]	0.033	0.15 [0.03; 0.77]	0.024
**Children**				
No	Ref		Ref	
Yes	0.12 [0.02; 0.93]	0.042	0.10 [0.01; 0.71]	0.022
**Professional stage**				
Family physician specialist	Ref		Ref	
Family physician trainee	0.01 [0.00; 0.10]	<0.001	0.02 [0.00; 0.12]	<0.001
**Years of experience**	0.97 [0.91; 1.03]	0.308	–	–
**Work-family conflict**	1.02 [0.76; 1.36]	0.893	–	–
**Physician burden**	0.83 [0.72; 0.95]	0.005	0.83 [0.75; 0.93]	0.001

The Hosmer-Lemeshow test indicates good model fit to the data (*p*-value = 0.406).

## 4 Discussion

Self-efficacy in managing multimorbidity was reported to be high in the current sample, with 67.6% of family physicians expressing confidence in delivering optimal healthcare to patients with multimorbidity. However, physician burden was associated with reduced odds of perceived self-efficacy in managing multimorbidity. In contrast, perceived organizational support showed no relationship with self-efficacy, and the negative association of work-family conflict disappeared in the multivariate analysis.

To our knowledge, this is the first study to assess self-efficacy in managing multimorbidity among Portuguese family physicians, and its comprehensive evaluation of multiple variables influencing self-efficacy. The use of multivariate analysis enhances the reliability of findings, while the inclusion of diverse demographic and professional characteristics strengthens their applicability. Nonetheless, this study has some limitations. The sample was obtained through a volunteer sampling approach and included a small number of family physicians; therefore, the results should be interpreted with caution and may not be generalizable to other clinical settings. Data were collected at a single time point, and thus the findings should not be interpreted as causal. The use of a self-administered questionnaire may also introduce measurement bias. Future research should aim to include larger, nationally representative samples.

The present study provides evidence of a negative relationship between physician burden and family physicians’ self-efficacy, aligning with previous findings among healthcare professionals (24). For instance, job stress among health teachers has been shown to increase burnout and reduce self-efficacy (31). Similarly, nursing students with low perceived stress levels exhibit significantly higher self-efficacy (32). Factors contributing to stress and diminished self-efficacy among healthcare professionals include workplace bullying, family stress, public misunderstanding, and limited opportunities for personal development (24).

The following demographic characteristics were associated with reduced odds of perceived self-efficacy in managing multimorbidity: being single, divorced, or widowed; having children; and being a family physician trainee.

It was previously suggested that the type of training received by healthcare professionals affects self-efficacy levels (33), so it is not surprising that being a family physician trainee was associated with reduced odds of perceived self-efficacy in the present study. These findings suggest that there is a need not only to address physician burden but also to provide targeted training in managing multimorbidity, which can contribute to improving physicians’ self-efficacy.

Another important finding is that family physicians with apparently less social support (being single, divorced, or widowed) have reduced odds of perceived self-efficacy. This could explain why work-family conflict does not significantly influence self-efficacy in the sample, as the absence of a strong social support system outweighs the benefits of fewer work-family conflicts. This result corroborate previous findings, which show that among nurse practitioners, social support from coworkers, family, and friends contributes significantly to self-efficacy (34). Additionally, social support from colleagues, spouses, friends, and support groups is known to help prevent burnout and other health issues in physicians (35).

Other authors have shown that the number of children is associated with an individual’s sense of self-efficacy, with evidence suggesting that having two children can enhance the self-efficacy of young parents (36). In contrast, the present study found that physicians with children had reduced odds of perceived self-efficacy in managing multimorbidity in their clinical practice. This divergence may be explained by the fact that, for physicians managing multiple chronic conditions in their patients, the presence of children may represent an added source of responsibility, burden and stress, potentially reducing their perceived control over professional capabilities.

Physicians, especially those in primary care, are often faced with the complex challenge of treating patients with multiple chronic conditions, which can lead to significant stress and burnout. The findings of the present study highlight the importance of addressing physician burden to improve perceived self-efficacy in managing multimorbidity. Therefore, efforts should focus on reducing this burden by alleviating workplace stress and providing targeted training in managing multimorbidity. Improving self-efficacy is expected to encourage physicians to engage in proactive, patient-centered care, leading to better health outcomes.

## Data Availability

The raw data supporting the conclusions of this article will be made available by the authors, without undue reservation.
